# Genetic code expansion enables plant-directed control of bacterial activity

**DOI:** 10.64898/2026.05.29.728790

**Published:** 2026-07-30

**Authors:** Vivian Zhong, Michaela A. Jones, Avaniek Cabales, Alice Gevorgyan, René Inckemann, Anna A. Johnson, Sumudu S. Karunadasa, Amanda M. Forti, Shou-Ling Xu, Aditya M. Kunjapur, Jennifer A. N. Brophy

## Abstract

Programmable control of microbial gene expression by plant hosts could enable a new generation of precision agricultural biotechnology. Here, using *O* -methyl-L-tyrosine (OMY) as a model compound, we establish non-canonical amino acids (ncAAs) as a tool for plant-based control of associated microbial activity. We use genetic code expansion to engineer OMY-dependent control of protein synthesis in the soil bacterium *Bacillus subtilis.* Then, we engineer agronomically diverse plants, including Arabidopsis, tomato and poplar, to biosynthesize OMY. We show that plant-derived OMY can stimulate gene expression in both model and wild soil bacteria while also demonstrating that inducible and tissue-specific expression of a single biosynthetic enzyme by the plant enables on-demand control over microbial activity. This work establishes ncAAs as a tool for programming plant-microbe partnerships.

## Full Text Availability

The license terms selected by the author(s) for this preprint version do not permit archiving in PMC. The full text is available from the preprint server.

